# Assessing the Mental Condition of Paramedics and Nurses during the SARS-CoV-2 Pandemic

**DOI:** 10.3390/jpm13010070

**Published:** 2022-12-28

**Authors:** Maksymilian Kilian, Piotr Gałecki, Agata Orzechowska

**Affiliations:** 1 WSRM Provincial Medical Emergency Station in Lodz, 90-001 Lodz, Poland; 2 Władysław Biegański Provincial Specialist Hospital in Lodz, 91-347 Lodz, Poland; 3 Department of Adult Psychiatry, Medical University of Lodz, 91-229 Lodz, Poland

**Keywords:** mental health, stress, COVID-19, pandemics, paramedics, nurses

## Abstract

Background: The COVID-19 pandemic has significantly affected many aspects of life. The aim of this study was to assess the mental state of medical personnel most involved with patients affected by SARS-CoV-2. Methods: The survey research was conducted between October 2021 and March 2022. The study group included 46 paramedics, 48 nurses, and 6 individuals from both professions, ranging in age from 21 to 67 years. Data were collected using paper questionnaires that contained 44 questions. Results: During the pandemic, respondents maintained good contact with their colleagues and were competent enough to help their patients. The main factors that influenced their stress or dissatisfaction were the number of patients and the number of tasks. The nurses and paramedics most frequently reported symptoms related to tension, insomnia, and problems with intellectual performance. The pandemic situation led to the abandonment of hobbies and deterioration of relationships with friends and family. Conclusion: As a result of high stress levels, paramedics and nurses frequently suffered from tension and insomnia. The factors described were associated with poorer well-being of the subjects in various functional areas, even before the pandemic period. This was mainly related to the large number of patients and the tasks. However, this work, as well as studies by other authors, come to alarming conclusions that should draw attention to the mental state of medical staff, as this is the group that is highly responsible for the medical care of patients, especially in such a difficult time as a pandemic.

## 1. Introduction

The first cases of infection with a new coronavirus variant, namely SARS-CoV-2 (severe acute respiratory syndrome coronavirus 2), were recorded in the city of Wuhan, China, in late 2019. In a short time, it covered the whole world and dramatically affected everyone’s life. Depending on the number of infections and the decisions made by national governments, the daily lives of citizens changed in different ways. To date, several publications have appeared dealing with the impact of the coronavirus pandemic on mental health [1,2,3,4], and the UN (United Nations) produced a special report on the subject [5]. In the report, the organization emphasized the importance of mental health for the proper functioning of society, presented statistics on the impact of SARS-CoV-2 on the population, identified the social groups most at risk of pandemic-related mental disorders, and proposed appropriate measures to combat the phenomenon.

The occupational groups studied were particularly exposed to the new infectious disease. Even before 2019, both paramedics [6,7,8,9] and nurses [10,11,12] were dealing with a variety of stressors in their professional lives. However, the outbreak of the SARS-CoV-2 pandemic may have further exacerbated mental health problems in these professions. This study focused specifically on people working in Lodz, Poland. According to the Polish EZOP I study [13], the province of Lodz had the highest prevalence rate for mental disorders compared to other provinces.

To date, a large number of studies on this topic have been produced and published worldwide [14,15,16,17,18]. A large proportion of these deal with health workers in Asia, particularly in China [17,18]. The authors of this study investigated stress responses and coping methods and analyzed the prevalence of worrisome psychosomatic symptoms related to stress exposure during the pandemic. In Poland, few papers have been published on this topic [19], but there are many articles dealing with stress and occupational burnout in nurses and paramedics before the declaration of the epidemic state in Poland that can serve as a reference.

We assumed that in the research conducted on a selected population of respondents, the period of the pandemic had a negative impact on the mental condition of paramedics and nurses.

## 2. Materials and Methods

A group of 100 respondents (Table 1) including 44 men and 56 women aged 21 to 67 years (M = 38.65, SD ± 12.57) participated in the study. A total of 48 respondents worked as nurses, 46 worked as paramedics, and 6 individuals practiced both professions. The mean number of years of work experience was 15.78 (SD ± 13.01). Respondents worked in hospitals (63 individuals), emergency medical services (29 individuals), or both (8 individuals).

The criterion for inclusion in the study group was to have worked as a paramedic or nurse in the Lodz region for at least 2 months during the epidemic state in Poland, i.e., between 20 March 2020, and 13 May 2022 (Regulation of the Minister of Health of 20 March 2020 on declaring a state of epidemic in the territory of the Republic of Poland). Such a period of time was sufficient to develop adverse symptoms, and it was necessary to exclude from the research group people who had just started working or who were only working in healthcare temporarily at the time.

A paper questionnaire prepared and developed by the authors of this article was used to conduct the study. The questionnaire was based on the Hamilton Anxiety Rating Scale (HAM-A) [20] and the Link Burnout Questionnaire (LBQ) by Massimo Santinello published by the Laboratory of Psychological Tests of the Polish Psychological Association [21].

The instrument consisted of four parts, each focusing on a different target. The first part, comprising 17 closed questions and 1 open question, examined the occurrence of selected stress-related phenomena at work. Respondents could rate the frequency of the listed sensations on a 5-point scale (never, rarely, sometimes, often, or always). The second part consisted of seven closed questions and examined the factors influencing the level of perceived stress on a 4-point scale (definitely not, rather not, rather yes, or definitely yes). The third part consisted of 11 closed and 1 open-ended question and examined the frequency of somatic and psychosomatic symptoms on a 5-point scale (never, rarely, sometimes, often, or always). The fourth part included 7 items and analyzed the intensity of negative stress-related phenomena on a 4-point scale (definitely not, rather no, rather yes, or definitely yes).

Questionnaires were completed on paper only in the presence of the authors who conducted the research or other selected individuals who assisted in data collection. 

Participation in the study was voluntary, and all respondents were informed of the aims and purpose of the data collection and gave consent. In addition, it was possible to withdraw from the study at any time. The study did not require approval from the Bioethics Committee because it did not meet the definition of a medical experiment. This article was written as part of a master’s thesis.

Analysis was performed using PQStat software (version 1.6.8). The following data were used for the analysis:

- Mann–Whitney U test to compare ordinal and quantitative variables (when the assumption of normal distribution was not met) between two groups;
- Spearman’s rank correlation coefficient, determined whether two ordinal variables are correlated;
- Friedman’s test for one-way analysis of variance with repeated measures by rank, which can be used to determine whether there are statistically significant differences between the ratings of each measure.

## 3. Results

### 3.1. Research Problem: Assessing the Frequency of Selected Phenomena and Sentiments during the SARS-CoV-2 Pandemic

The frequency of the selected feelings was examined on a scale from 1 (never) to 5 (always). The descriptive statistics for each feeling can be found in Table 2. In addition, the table shows the results of the analysis of variance with repeated measures by rank (Friedman test).

The analysis (Table 2) confirmed differences in the frequency of each feeling between subjects (*p* < 0.001). The sentiment most frequently reported by respondents was the statement, “*You got along well with other employees at work*”; three-quarters of respondents chose 4 or 5, and one-quarter of respondents gave the highest value (Q25 = 4; Me = 4; Q75 = 5). This statement was evaluated similarly to the statement, “*You felt you were competent enough to help your patients*”. Using the Dunn–Bonferroni post hoc test, no statistically significant differences were found between the two statements (*p* > 0.05). All other statements were rated lower (indicated by lower frequency of sentiments) than the statement, “*You got along well with other employees at work*”. Regarding the statement, “*You felt you were competent enough to help your patients*”, three quarters of respondents indicated a response of 4 or 5 (Q25 = 4), but the group of those who chose the highest rating was smaller than for the for the most stated sentiment (Q75 = 4; Max = 5). These results were not statistically significantly different from those for the next statement, i.e., “*You felt that you were able to properly organize the work in your position*” (Q25 = 3; Me = 4; Q75 = 4).

### 3.2. Research Problem: Analysis of Factors Affecting Levels of Perceived Stress and Dissatisfaction

The influence of each factor on respondent stress or dissatisfaction was examined on a scale of 1 (definitely no) to 4 (definitely yes). The descriptive statistics for each factor are shown in the table below. In addition, the table provides the results of the analysis of variance with repeated measures by rank (Friedman test).

The test result (Table 3) is statistically significant (*p* < 0.001), which means that there are differences in the evaluation of the various factors that affect the level of perceived stress or dissatisfaction. Based on descriptive statistics and the Dunn–Bonferroni post hoc test, it was found that the factors that most influenced the level of perceived stress or dissatisfaction were the number of patients and the number of tasks; three-quarters of the respondents answered rather yes or definitely yes. The results for the number of patients and the number of tasks were not statistically significantly different (the result of the post hoc test between the two factors was not statistically significant). This was followed by salary as an important factor, to which half of the respondents answered rather yes or definitely yes. This factor was rated lower than the number of patients (*p* < 0.05) was but comparable to the number of tasks (*p* > 0.05).

### 3.3. Research problem: Assessing the Prevalence of Adverse Somatic and Psychosomatic Symptoms Related to Stress or Dissatisfaction in the Workplace

The frequency of the selected symptoms was examined on a scale of 1 (never) to 5 (always). The descriptive statistics for each symptom are shown in Table 4. In addition, the table provides the results of the analysis of variance with repeated measures by rank (Friedman test).

The test result (Table 4) is statistically significant (*p* < 0.001), which means that there are differences in terms of indications for individual symptoms. The highest scores were found for tension, insomnia, and intellectual performance (Me = 3). Based on the Dunn–Bonferroni post hoc test, no statistically significant differences (*p* > 0.05) were found between these symptoms. For tension and insomnia, differences were found for all other symptoms. On the other hand, scores for intellectual performance (Q25 = 2; Me = 3; Q75 = 3) were also not statistically significantly different from those for depressed mood (Q25 = 2; Me = 3; Q75 = 3) and anxious mood (Q25 = 2; Me =2.5; Q75 = 3) (*p* > 0.05).

### 3.4. Research Problem: Analysis of the Presence of Harmful Behaviors in Response to Stress among Survey Respondents

The occurrence of selected behaviors was examined on a scale of 1 (definitely no) to 4 (definitely yes). The descriptive statistics for each behavior can be found in the Table 5. In addition, the table provides the results of the analysis of variance with repeated measures by rank (Friedman test).

The test result (Table 5) is statistically significant (p < 0.001), which means that there are differences in the frequency of certain behaviors during the pandemic. Based on the descriptive statistics and the Dunn–Bonferroni post hoc test, the most frequent behaviors of the subjects include giving up their hobbies and deteriorating quality of relationships with friends; the ratings of these behaviors do not differ, as shown by the values of the descriptive statistics (Q25 = 2; Me = 2; Q75 = 3) and the statistically non-significant result of the post hoc test (p > 0.05). References to the listed behaviors were statistically more frequent than references to the other behaviors, with the exception of deterioration in relationships with family (Q25 = 1; Me = 2; Q75 = 3). The next behaviors indicated were more frequent consumption of alcoholic beverages, followed by increased cigarette smoking.

### 3.5. Dependence of Results on Variables (Age, Length of Service, Gender, and Occupation)

#### 3.5.1. Selected Sentiments and Phenomena Related to the Workplace

The analysis examined whether there was a relationship between the variables and age, length of service, gender, and occupation and the frequency of selected feelings and phenomena at work.

#### Age

Frequency correlated with age for the following sentiments:

- You felt that you were able to properly organize the work in your position (*r* = 0.361; *p* < 0.001). The correlation was positive, meaning that the older the respondents were, the more often they felt they could organize the work in their position adequately (the correlation was moderately strong);
- Breaks at work were enough for you to rest (*r* = 0.217; *p* = 0.031). The older the respondents were, the more often they indicated that work breaks were sufficient for them to rest (the correlation was weak);
- You felt you were competent enough to help your patients (*r* = 0.224; *p* = 0.026). The correlation was positive; higher age was associated with a more frequent belief that respondents were competent enough to help patients (the correlation was weak);
- Statistically insignificant results (*p* > 0.05) were recorded for the other feelings, meaning that the results were not correlated with age.

#### Length of Service

Frequency correlated with length of service for the following sentiments:

- You felt that you were able to properly organize the work in your position (*r* = 0.229; *p* = 0.003). The correlation was positive, meaning the more experienced the respondents were, the more often they felt that they could organize the work in their position adequately (the correlation was weak);
- Breaks at work were enough for you to rest (*r* = 0.238; *p* = 0.018). The more experienced the respondents were, the more often they indicated that work breaks were sufficient for them to recover (the correlation was weak).
- You felt you were competent enough to help your patients (*r* = 0.253; *p* = 0.012). The correlation was positive; more experience was associated with a more frequent belief that respondents were competent enough to help patients (the correlation was weak).

There were statistically insignificant results (*p* > 0.05) for the other sentiments, meaning that the results were not correlated with experience (length of service).

#### Gender

Statistically non-significant results (*p* > 0.05) were reported for each sentiment. This means that the frequency of each feeling did not depend on the gender of the subjects.

#### Occupation

People with both professions were excluded from the analysis - due to a too small group size it was not possible to include them in the analysis.

**Figure 1:**
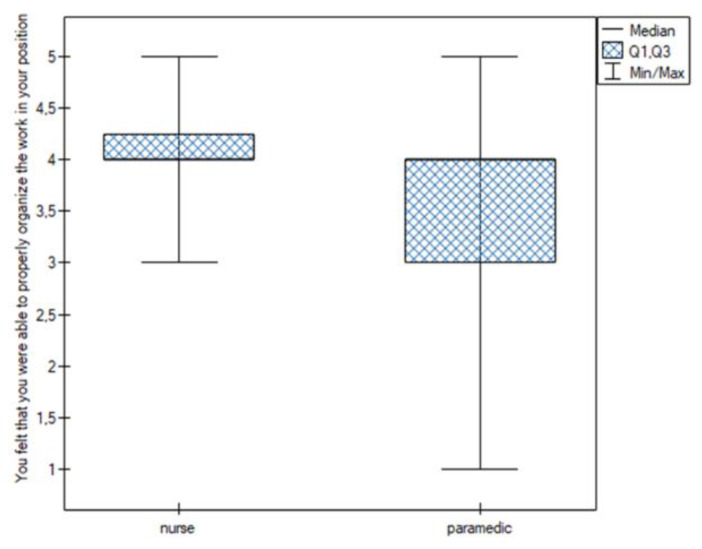
Work organization.

**Figure 2:**
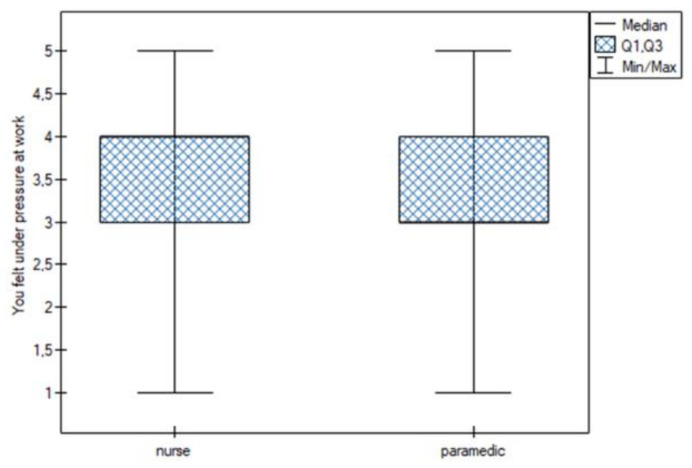
More difficult patients.

**Figure 3:**
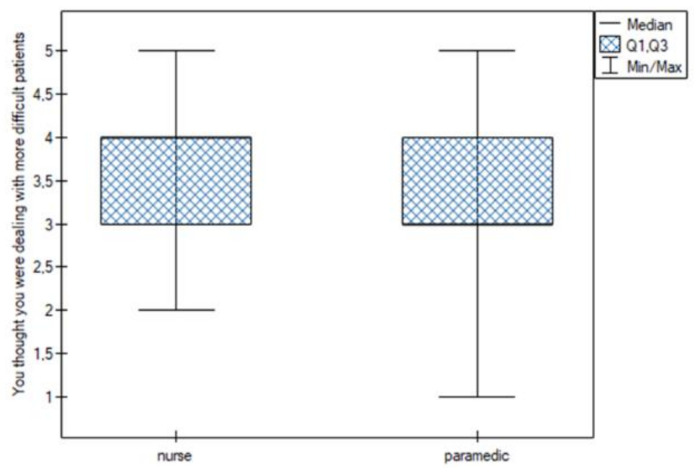
Working under pressure.

**Figure 4:**
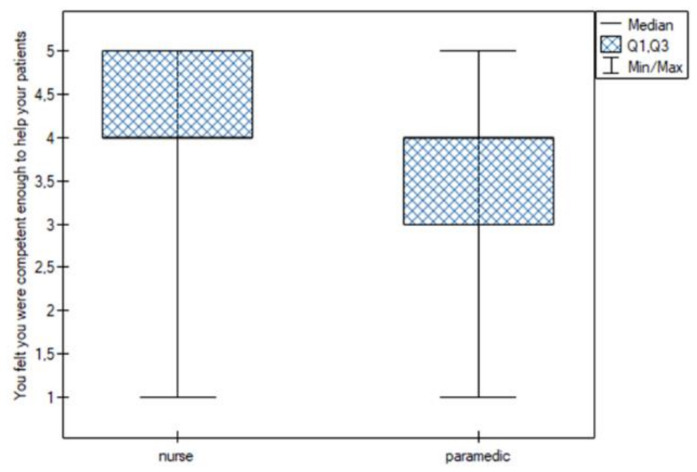
Sufficient competence.

Statistically significant differences were noted for the following sentiments:

- You felt that you were able to properly organize the work in your position. This feeling was more common among nurses (Q25 = Me = 4; Q75 = 4.25) than among paramedics (Q25 = 3; Me = 4; Q75 = 4) (Figure 1);
- You thought you were dealing with more difficult patients. This feeling was more common among nurses (Me = 4) than among paramedics (Me = 3) (Figure 2);
- You felt under pressure at work. This feeling was more common among nurses (Me = 4) than among paramedics (Me = 3) (Figure 3);
- You felt you were competent enough to help your patients. This feeling was more common among nurses (Q25 = Me = 4; Q75 = 5) than among paramedics (Q25 = 3; Me = 4; Q75 = 4) (Figure 4).

#### 3.5.2. Factors Affecting the Level of Perceived Stress or Dissatisfaction

##### Age

The following table (Table 6) shows the results of the analysis based on Spearman’s rank correlation coefficient. The purpose was to determine whether the factors reported by the respondents that influence the level of perceived stress and dissatisfaction are correlated with the age of the subjects.

The older the respondents were, the less frequently they reported things such as availability of personal protective equipment (*r* = −0.202; *p* = 0.045), availability of medical equipment (*r* = −0.330; *p* < 0.001), and number of patients (*r* = −0.228; *p* = 0.023). The correlations were negative, and their strength was weak. There was no correlation with age for the other factors (*p* > 0.05).

##### Length of Service

The following table (Table 7) shows the results of the analysis based on Spearman’s rank correlation coefficient. The purpose was to determine whether the factors reported by the respondents that influence the level of perceived stress and dissatisfaction are correlated with the length of service of the subjects.

The more experienced the subjects were, the less frequently they reported the availability of medical equipment (*r* = −0.266; *p* = 0.008) and the number of patients (*r* = −0.224; *p* = 0.026). These correlations were weak. There was no correlation with work experience (length of service) for the other variables.

##### Gender

During the study, an analysis based on the Mann–Whitney U test was performed to verify the presence of gender-related differences.

The rating of factors affecting the level of perceived stress or dissatisfaction did not differ according to the gender of the subjects (*p* > 0.05).

##### Profession

Opinions about factors influencing perceived stress or dissatisfaction varied by occupational group in relation to salary (U = 832.00; *p* = 0.030). This theme was mentioned more frequently by nurses (Q25 = 2; Me = 3; Q75 = Max = 4) than by paramedics (Q25 = 2; Me = Q75 = 3). There were no differences between occupations for the other factors (*p* > 0.05).

#### 3.5.3. Somatic and Psychosomatic Symptoms

##### Age

An analysis based on Spearman’s rank correlation coefficient was performed to determine whether the frequency of each symptom was correlated with the age of the subjects.

The incidence was correlated with age for the following symptoms:

- Anxious mood (*r* = 0.200; *p* = 0.047): the older the subjects were, the more often they reported an anxious mood (the correlation was positive and weak);
- Somatic zone (senses) (*r* = 0.337; *p* = 0.001): the correlation was moderately strong and positive (the older the subjects were, the more often they indicated the area in question);
- Cardiovascular system (*r* = 0.250; *p* = 0.013): the correlation was weak and positive (the older the subjects were, the more often they indicated the cardiovascular system);
- Respiratory system (*r* = 0.231; *p* = 0.021): the correlation was weak and positive (the older the subjects were, the more often they indicated the respiratory system);
- Digestive tract (*r* = 0.205; *p* = 0.042): the correlation was weak and positive (the older the subjects were, the more often they indicated the digestive tract).

##### Length of Service

The table below shows the results of the analysis based on Spearman’s rank correlation coefficient to determine whether the frequency of each symptom in the subjects was correlated with their length of service.

Frequency was correlated with the length of service for the following symptoms:

- Anxious mood (*r* = 0.209; *p* = 0.039): the more experienced the subjects were, the more often they indicated an anxious mood (the correlation was positive and weak);
- Somatic zone (senses) (*r* = 0.327; *p* = 0.001): the correlation was moderately strong and positive (the more experienced the subjects were, the more often they indicated the area in question);
- Cardiovascular system (*r* = 0.232; *p* = 0.021): the correlation was weak and positive (the more experienced the subjects were, the more often they indicated the cardiovascular system);
- Respiratory system (*r* = 0.215; *p* = 0.034): the correlation was weak and positive (the more experienced the subjects were, the more often they indicated the respiratory system);
- Digestive tract (*r* = 0.202; *p* = 0.046): the correlation was weak and positive (the more experienced the subjects were, the more often they indicated the digestive tract).

##### Gender

The following table (Table 8) shows the results of the analysis based on the Mann–Whitney U test to verify the presence of gender-related differences.

Subjects’ responses to the questions about pandemic symptoms differed by gender for all aspects studied except intellectual performance. For each symptom, females reported a higher prevalence than males. 

##### Profession

The following table (Table 9) shows the results of the analysis based on the Mann–Whitney U test to verify the existence of differences by occupation.

Occupation differentiated the subjects’ responses regarding pandemic symptoms for all aspects studied except intellectual performance. For each symptom, the nurses reported a higher prevalence than the paramedics. 

#### 3.5.4. Harmful Behaviors Associated with Stress and Dissatisfaction

Analyses were performed using the Mann–Whitney U test to test for the presence of differences by age, seniority, gender, and occupation.

#### Age

A correlation of prevalence with age for risky sexual behavior was found for the behaviors studied (*r* = −0.218; *p* = 0.030). The correlation was negative, i.e., the younger the subjects were, the more frequently they reported engaging in risky sexual behavior. There was no correlation with age for the other behaviors (results were not statistically significant; *p* > 0.05).

#### Length of Service

For the behaviors studied, a correlation was found between the frequency of their occurrence and the length of service for risky sexual behavior (*r* = −0.234; *p* = 0.020). The correlation was negative, meaning that the shorter the subjects’ length of service, the more frequently they reported engaging in risky sexual behavior. No correlation with length of service was found for the other behaviors (results were not statistically significant; *p* > 0.05).

#### Gender

**Figure 5:**
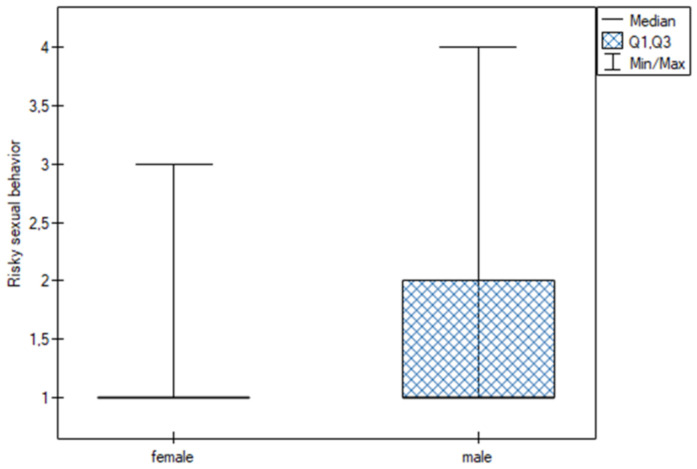
Risky sexual behavior.

Analysis of data on specific behaviors by gender revealed statistically significant differences in risky sexual behaviors (U = 956.00; *p* = 0.003). These behaviors were reported more frequently by males (Q25 = 1; I = 1; Q75 = 2) than by females (Q25 = 1; I = 1; Q75 = 1). No gender differences were found for the other behaviors (Figure 5).

#### Occupation

Analysis of data on specific behaviors by occupation revealed statistically significant differences with respect to risky sexual behaviors (U = 956.00; *p* = 0.003). These behaviors were reported more frequently by paramedics (max = 4) than by nurses (max = 3). No differences were found between the occupations for the other behaviors.

## 4. Discussion

The results of the survey show many problems associated with healthcare work, not only during the pandemic. In the first part of the survey, we can see that physical exhaustion, pressure, tension, and disappointment with work were present in almost all respondents but with varying frequency. However, it seems reassuring that the negative feelings are balanced by the occurrence of many positive feelings with relatively high frequency. Particularly noteworthy is the frequently mentioned confidence in one’s professional competence and ability to organize one’s job despite the difficult conditions associated with the pandemic, as well as the very rare thoughts of turning back the clock and changing jobs. Another balancing aspect of negative experiences is the repeatedly provided response regarding contact with colleagues; these relationships may have proven important in coping with the new, difficult situation.

The factors cited by survey participants as having the greatest impact on the level of perceived stress and dissatisfaction were the number of patients and the tasks. Based on the context of the study, although it was not stated whether there were more or fewer, it can be surmised that there were more than before the outbreak of the pandemic, which could only have had a negative impact on the mental health of medical staff. This could also indicate that the number of patients before the pandemic was also very high and was already affecting stress and dissatisfaction. It is also worth mentioning the next most common factor influencing stress and dissatisfaction, namely financial compensation. It should be noted that paramedics and nurses in Poland who came into contact with patients diagnosed or suspected of having coronavirus were entitled to salary supplements, which were usually 100% of their basic salary [22]. However, the questionnaire does not provide us with an answer to the question of whether the lack of additional compensation received by some employees discouraged them from taking the risk of working near infected patients or whether the allowance compensated for the harsh experience of the pandemic. The same research issue was raised in a study by Charzyńska-Gaula et al. [12], in which low salary was the factor most frequently cited by respondents as a cause of high levels of stress. 

Somatic and psychosomatic symptoms discussed in this study include insomnia (such as difficulty falling asleep, irregular sleep, unsatisfactory sleep, fatigue upon waking, nightmares, or night terrors), tension (such as feelings of tension, fatigue, violent reactions, tendency to cry, body tremors, feelings of restlessness, and inability to rest/relax), and symptoms related to intellectual performance (difficulty concentrating and poor memory). Whereas the first two symptoms occur in medical personnel working under highly stressful conditions regardless of the ongoing pandemic, difficulty concentrating and poor memory seem to be of particular concern among paramedics and nurses, as such symptoms can lead to errors and adverse events, affecting patients’ lives and health. 

The term ‘post-COVID brain fog’ has already been mentioned in the literature and can summarize such a clinical picture [23,24,25]. In 2021, Hellmuth et al. [24] described a study of two patients who had contracted the new coronavirus variant but were not hospitalized for it. Results of routine testing were not abnormal, but more detailed testing showed deficits in working memory and cognitive function. In addition, a cohort observational study of 100 subjects who were in recovery (COVID -19) identified 14 non-hospitalized subjects who had uninterrupted cognitive impairments that lasted at least 98 days. In addition, Theoharides et al. [25] described cognitive dysfunction and fatigue in patients with long COVID. The authors compared the clinical picture and etiopathogenesis with patients who had received chemotherapy (’chemo-fog’) and with patients with myalgic encephalomyelitis/chronic fatigue syndrome (ME/CFS) or mast cell activation syndrome (MCAS). Cognitive functions are significantly related to the way reality is interpreted and, consequently, to the occurrence of emotional states and sustained mood.

In this section, the last follow-up question, which dealt with the prevalence of the listed symptoms before the introduction of the epidemic state in Poland, proved to be crucial. The results clearly show that most respondents felt that their symptoms had worsened during the pandemic (41%), but when asked in an open-ended question to name or select specific symptoms from those listed, 84% were unable to do so. There may be several reasons for this situation. The most probable is that the high stress level and constant readiness of the respondents did not allow for free reflection on their mental state and all related problems relative to the period before the introduction of the epidemic state in Poland. This resulted in difficulty in remembering the specific symptoms that had accompanied them before. Another likely explanation for these differences in responses is a misunderstanding or haste in completing the survey that was not communicated to the investigator, resulting in a failure to provide detailed responses. To determine the cause of this problem, the survey participants would need to be interviewed in depth. This could be a limitation of the present work.

The analysis of the last part of the questionnaire revealed that the epidemiological situation, as well as the negative situations, feelings, and symptoms that followed, had the greatest impact on interpersonal relationships and only secondarily on extra-occupational interests and hobbies. Such disintegration processes in interpersonal relationships could be due to the danger posed by the transmission of the coronavirus to close contacts, as well as to the frequent compliance with quarantine by personnel who were released from quarantine only after a certain period of the pandemic. Abandonment of hobbies and interests and deterioration of contact with family and friends could also be attributed to the high number of hours worked, especially among staff who had more than one job or who worked more hours in their only job than a full-time position warranted. Such excessive workload that increased the risk of burnout could have had similar effects even in the absence of a global pandemic, but this hypothesis requires detailed analysis and comparison with prepandemic statistics.

The literature on this topic is not extensive, and the conclusions drawn from the observations made are consistent with other studies on related topics and with information available in the literature.

Barszczak [6] analyzed stress in the paramedic profession. The results show that paramedics consider themselves stress-resistant individuals who can handle difficult situations and emergencies. The biggest stressors for them are the high level of responsibility and time pressure. Nevertheless, the respondents indicated that they believe that they are able to deal with stress appropriately. The results suggest that stress negatively affects the respondents’ ability to concentrate and leads to fatigue and irritability, but they could not indicate how often they experience such stress. The most frequently chosen methods of coping with stress included hobbies and passions, as well as talking with close contacts.

In 2015, Nowicki et al. [8] conducted a study on how paramedics cope with stress at work. A proportion of 92% of respondents admitted to experiencing stress related to their work. Respondents used two coping strategies to deal with stress, namely the active coping model, in which they take action to improve their situation, and the acceptance model, which involves accepting the current situation and learning to live with it. The author of the article also emphasized the major role that relationships with loved ones and family, and thus the ability to talk to others, play in the process of coping with stress.

In a 2015 study conducted by Rasmus et al. [9] on the effects of experienced stress on risky behaviors in a group of emergency medical technicians (EMT), the results showed that 82.14% of the 140 county and provincial emergency department personnel surveyed experienced high levels of work-related stress. The most common risky behaviors were alcohol consumption (95%), dangerous traffic behaviors (55%), and smoking (45.5%). In addition, a relationship was found between the level of perceived stress and the occurrence of risky behaviors. The authors concluded that because of the high level of perceived stress, appropriate methods and techniques should be used to reduce the negative effects of experiencing traumatic events.

In 2015, Kędra and Nowocień [11] investigated the relationship between stressors and burnout risk in nurses. During data collection, 200 individuals completed the survey. A total of 129 individuals chose the highest score when asked whether salaries are inadequate relative to the demands of the job, and 100 respondents gave the highest score for an unacceptable ratio of salaries to other occupations. In addition, it was concluded that an important factor that causes job dissatisfaction and increases the risk of burnout is being overloaded with job tasks. The most commonly cited stressors in nurses’ jobs are an excessive number of tasks, responsibility for another person’s health, and dissatisfaction and resentment from patients and their families.

In a paper published in 2016 by Charzyńska-Gaula et al. [12], the authors analyzed the causes of occupational stress perceived by nurses. Most of the 278 nurses surveyed (85%) were satisfied with their work, but as many as 95% said that the nursing profession was stressful. A proportion of 56.1% of respondents indicated that they were exposed to occupational stress on a daily basis. The most frequently cited factors that caused significant levels of stress were low pay and sudden deterioration of patients’ condition and the need for resuscitation.

Antonijevic et al. [15] conducted a study with 1678 participants to assess their stress levels by comparing medical staff working on the ‘frontline’ with those working on the ‘second line’ in health care during the COVID-19 pandemic in Serbia. The results of their study showed that frontline staff had twice the risk of developing severe anxiety-related symptoms. In addition, the study authors concluded that healthcare workers who work directly with patients had higher levels of stress, anxiety, and depressive symptoms. They also called for the implementation of appropriate psychological support and stress management interventions for these employees.

In 2020, Cai et al. [17] investigated the effects of stress on healthcare workers in Hunan Province, China, as well as coping strategies among healthcare workers dealing with the new variant of coronavirus. A total of 534 workers completed the relevant questionnaires. Results indicated that they felt professionally and socially obligated to work longer hours and feared for their safety and the safety of their loved ones, and that emerging reports of COVID-19-related deaths had negative psychological effects. In addition, it was found that the availability of appropriate procedures in case of infection, specialized equipment, appreciation of the efforts by hospital management and the government, and a decrease in the number of coronavirus cases led to a noticeable improvement in psychological status. According to the authors, medical personnel in Hunan province experienced a significant increase in stress levels due to the SARS-CoV-2 outbreak in the neighboring province of Hubei. In addition, they pointed out that continued recognition of medical personnel by hospital management and the government, provision of appropriate infection guidelines, specialized equipment, and adequate facilities for COVID-19-infected patients would encourage medical personnel to make the effort to combat outbreaks in the future.

## 5. Conclusions

This paper does not fully exhaust the issues raised. Instead, it presents a very interesting observation about the impact of the pandemic on medical professions that had already been exposed to high stress levels for a long time. The state of the epidemic in Poland provided medical personnel with partial compensation for their distress in the form of special monetary allowances, but this did not offset all of the negative effects. A large number of patients, a flood of tasks, and inadequate pay created many problems in the psyche of medical personnel, but they also reinforced the preexisting negative symptoms and feelings that had accompanied them before the pandemic. In addition, paramedics and nurses themselves were exposed to the stress of fearing for their own lives and health. The current study provides an introduction to this topic and may serve as a basis for further study and research on this and related topics that will contribute to the discussion of psychological stress among medical personnel. In such a situation, it is desirable to provide psychological support to this professional group, to improve working conditions (which is not always possible in an exceptional situation such as an epidemic), and to provide adequate compensation to these individuals for the harmful environment in which they work, as well as to establish appropriate psychological support mechanisms. However, it is encouraging to see that the interviewed medical personnel believe in their knowledge, they are able to manage and organize their workplace under difficult conditions, they maintain close relationships with their colleagues, they are attached to their profession, and they do not seem to have any desire to change it.

## Figures and Tables

**Table 1:** Research group description.

**Variable: Gender**	**Number**	**Percentage**
female	56	56%
male	44	44%
**Variable: Profession**	**Number**	**Percentage**
nurse	48	48%
nurse/paramedic	6	6%
paramedic	46	46%
**Variable: Work**	**Number**	**Percentage**
Medical emergency station	29	29%
Medical emergency station/hospital	8	8%
Hospital	63	63%
**Variable: Age**		
Arithmetic mean	38.65	
Standard deviation	12.57	
Minimum	21	
Maximum	67	
**Variable: Experience**		
Arithmetic mean	15.78	
Standard deviation	13.01	
Minimum	1	
Maximum	41	

**Table 2:** Subjective feelings and symptoms reported by the research participants during the epidemic period.

How Often Did You Experience the Following Sentiments during the Pandemic?	M	Me	SD	Min	Max	Q25	Q75
You felt that work was exhausting you physically	3.61	4	0.85	1	5	3	4
You felt that you were able to properly organize the work in your position	3.81	4	0.83	1	5	3	4
You thought you were dealing with more difficult patients	3.57	4	0.88	1	5	3	4
You were as passionate about your work as before	3.2	3	1.19	1	5	2	4
You felt under pressure at work	3.49	4	1.01	1	5	3	4
You got along well with other employees at work	4.07	4	0.71	2	5	4	5
You felt tense at work	3.05	3	0.96	1	5	2.75	4
When you were working, you felt full of energy	3.04	3	0.93	1	5	2	4
You felt disappointed with your work	2.75	3	0.95	1	5	2	3
Breaks at work were enough for you to rest	2.37	2	1.05	1	5	2	3
Your work made you feel active	3.2	3	1.16	1	5	2	4
You felt safe at work	3.16	3	1.16	1	5	2	4
You felt you were competent enough to help your patients	3.94	4	0.87	1	5	4	4
You thought about turning back the clock and choosing a different profession	2.39	2	1.17	1	5	1	3
The number of days off allowed you to fully recover	2.59	2	1.06	1	5	2	3
You doubted that what you were doing had any value	2.68	3	1.06	1	5	2	3
**Test result**							
T1	385.27						
*p*	<0.001						
T2	34.22						
*p*	<0.001						

M—mean; Me—median; SD—standard deviation; Min—minimum value; Max—maximum value; Q25—lower quartile; Q75—upper quartile; *p*—significance; T1—Friedman statistics; T2—Iman–Davenport statistics.

**Table 3:** Factors related to the level of perceived stress.

Did the Following Factors Affect Your Level of Perceived Stress or Dissatisfaction?	M	Me	SD	Min	Max	Q25	Q75
Form of employment (hours, type of contract)	2.12	2	0.87	1	4	1	3
Salary	2.91	3	0.87	1	4	2	4
Employer’s financial situation	2.05	2	0.87	1	4	1	3
Availability of personal protective equipment (gloves, coveralls, etc.)	2.13	2	1.04	1	4	1	3
Availability of medical equipment	2.29	2	0.98	1	4	2	3
Number of duties	3.08	3	0.82	1	4	3	4
Number of patients	3.35	3	0.74	1	4	3	4
**Test result**							
T1	234.77						
*p*	<0.001						
T2	63.64						
*p*	<0.001						

M–mean; Me—median; SD—standard deviation; Min—minimum value; Max—maximum value; Q25—lower quartile; Q75—upper quartile; *p*—significance; T1—Friedman statistics; T2—Iman–Davenport statistics.

**Table 4:** Somatic and psychosomatic symptoms reported by the respondents.

How Often Did You Experience the Following Symptoms during the Pandemic?	M	Me	SD	Min	Max	Q25	Q75
Anxious mood	2.56	2.5	1.18	1	5	2	3
Tension	2.79	3	1.20	1	5	2	4
Insomnia	2.84	3	1.21	1	5	2	4
Intellectual functioning	2.76	3	1.00	1	5	2	3
Depressive mood	2.59	3	1.06	1	5	2	3
Somatic zone (muscles)	2.19	2	1.14	1	5	1	3
Somatic zone (senses)	2.12	2	1.14	1	5	1	3
Cardiovascular system	1.95	1.5	1.15	1	5	1	3
Respiratory system	1.8	1	1.10	1	5	1	3
Digestive system	2.21	2	1.19	1	5	1	3
**Test result**							
T1	194.18						
*p*	<0.001						
T2	27.26						
*p*	<0.001						

M—mean; Me—median; SD—standard deviation; Min—minimum value; Max—maximum value; Q25—lower quartile; Q75—upper quartile; *p*—significance; T1—Friedman statistics; T2—Iman–Davenport statistics.

**Table 5:** Harmful behaviors.

Did You Notice the Following Behaviors in Yourself during the Pandemic?	M	Me	SD	Min	Max	Q25	Q75
More frequent use of alcoholic beverages	1.91	2	0.90	1	4	1	2
Increased number of cigarettes smoked	1.82	1	1.10	1	4	1	3
More frequent use of psychoactive substances	1.21	1	0.57	1	4	1	1
Risky sexual behavior	1.22	1	0.54	1	4	1	1
Deterioration of relationships with family	2.14	2	0.91	1	4	1	3
Deterioration of relationships with friends	2.33	2	1.00	1	4	2	3
Giving up your hobby	2.4	2	1.02	1	4	2	3
**Test result**							
T1	203.09						
*p*	<0.001						
T2	50.66						
*p*	<0.001						

M—mean; Me—median; SD—standard deviation; Min—minimum value; Max—maximum value; Q25—lower quartile; Q75—upper quartile; *p*—significance; T1—Friedman statistics; T2—Iman–Davenport statistics.

**Table 6:** Factors affecting stress according to age.

Did the Following Factors Affect Your Level of Perceived Stress or Dissatisfaction?	*r*	*p*
Form of employment (hours, type of contract); age	−0.087	0.394
Salary; age	0.112	0.268
Employer’s financial situation; age	–0.004	0.971
Availability of personal protective equipment (gloves, coveralls, etc.); age	−0.202	0.045
Availability of medical equipment; age	−0.330	0.001
Number of duties; age	−0.077	0.447
Number of patients; age	−0.228	0.023

*r*–Spearman’s rank correlation coefficient; *p*—significance.

**Table 7:** Factors affecting stress according to experience.

Did the Following Factors Affect Your Level of Perceived Stress or Dissatisfaction?	*r*	*p*
Form of employment (hours, type of contract); experience	−0.043	0.672
Salary; experience	0.078	0.448
Employer’s financial position; experience	0.061	0.548
Availability of personal protective equipment (gloves, coveralls, etc.); experience	−0.142	0.162
Availability of medical equipment; experience	−0.266	0.008
Number of duties; experience	−0.057	0.581
Number of patients; experience	−0.224	0.026

*r*–Spearman’s rank correlation coefficient; *p*—significance.

**Table 8:** Somatic and psychosomatic symptoms according to gender.

How Often Did You Experience the Following Symptoms during the Pandemic?	Female	Male	Test Result	
	Me	Me	U	*r*
Anxious mood	3	2	861.5	0.008
Tension	3	2	796	0.002
Insomnia	3	2	814	0.003
Intellectual functioning	3	3	1087	0.288
Depressive mood	3	2	937.5	0.034
Somatic zone (muscles)	2	1	781.5	0.001
Somatic zone (senses)	2	1	590	<0.001
Cardiovascular system	2	1	778	0.001
Respiratory system	2	1	869	0.005
Digestive system	2.5	1	695	<0.001

Me—median; U—Mann–Whitney U—test statistic; *p*—significance.

**Table 9:** Somatic and psychosomatic symptoms according to occupation.

How Often Did You Experience the Following Symptoms during the Pandemic?	Nurse	Paramedic	Test Result	
	Me	Me	U	*p*
Anxious mood	3	2	704	0.002
Tension	3	2.5	733	0.004
Insomnia	3	3	787.5	0.014
Intellectual functioning	3	3	1005	0.432
Depressive mood	3	2	834	0.034
Somatic zone (muscles)	2	2	886.5	0.088
Somatic zone (senses)	2	1	553	0.000
Cardiovascular system	2	1	713.5	0.002
Respiratory system	2	1	776	0.006
Digestive system	3	1	658	0.000

Me–median; U - Mann–Whitney U test statistic; *p*—significance.

## Data Availability

Data are available upon request from the corresponding author.
